# Microclimatic Impact Analysis of Multi-Dimensional Indicators of Streetscape Fabric in the Medium Spatial Zone

**DOI:** 10.3390/ijerph16060952

**Published:** 2019-03-16

**Authors:** Yunfang Jiang, Xuemei Han, Tiemao Shi, Danran Song

**Affiliations:** 1 Center for Modern Chinese City Studies, School of Urban and Regional Science, East China Normal University, Shanghai 200062, China; hxm511513@126.com (X.H.); danransong@126.com (D.S.); 2 Research Center for Eco Civilization, Shanghai Institute of Eco-Chongming, Shanghai 200062, China; 3 Institute for Innovation and Strategic Studies, East China Normal University, Shanghai 200062, China; 4 Institute of Spatial Planning and Design, Shenyang Jianzhu University, Shenyang 110168, China

**Keywords:** urban streetscape fabric, microclimate, street morphological index, ENVI-met, Shanghai

## Abstract

Different historical backgrounds and planning ideas have created different urban streetscape fabrics. The patterns of the streetscape fabric have affected urban microclimate factors and formed a unique local microclimate. This paper simulated the microclimatic effects in four study areas with different streetscape fabrics in Shanghai to compare the microclimatic conditions with a system of multi-dimensional street morphological indices using ENVI-met 4.3 software. At the street network fabric level, the results showed that streets with a south–north orientation, a small junction spacing, and a street network with better connectivity were conducive to mitigation of the air temperature heating intensity in the street space and improving the ventilation effect; at the street-site level: The indices of Build-to-line ratio (BL), Height-width ratio (H/W), and Sky view factors (SVF) played different roles that affected the distribution characteristics of the microclimate factors. The BL value of the streets between 0.5 and 0.8 generally had a positive relationship with the air temperature. The SVF value of the streets was positively correlated with the microclimate index, while the H/W values were negatively correlated with them. The morphological indicators of different levels also had a synergistic effect on the microclimatic impact of the street space fabric. This comparative analysis of microclimatic characteristics at the medium spatial scale will provide useful suggestions for urban climate adaptability in urban spatial morphology optimization in future urbanization development.

## 1. Introduction

Cities are composed of a series of different urban interfaces with continuity and diversity. With respect to fractal theory, the various interfaces of this diversity have different levels that constitute a dynamic spatial form of urban areas. Different historical contexts and different spatial scales of continuous areas are linked through the network and ultimately form the contemporary urban spatial fabric under the influence of the backgrounds of different eras. These different types of urban forms and structures create the complexity of the urban system. Every city depends on the needs of form, composition, and substructure to determine the success or failure of the city [1]. The urban built form is composed of a series of spatial factors or urban design variables, which has modified the urban climate and energy balance in cities. In recent years, significant progress has been made in the representation variables of urban spatial pattern, which can be used more systematically to describe urban spatial form and structure [2,3,4]. Spatial parameters can describe existing structural characteristics of different urban spatial fabric; for instance, to highlight the common features of urban spatial structure, thus supporting the division of urban typologies [5,6,7,8]. These parameters can form a set of decision-making tools to help planners intuitively and effectively to make a guideline for urban planning and design [3].

At the macro scale, the influence of the characteristics and forms of urban underlying surface on the urban local climate has been studied in depth for many years. Air temperature of the surface layer is greatly affected by the urban morphology, while the internal temperature in urban canopies changes dramatically [9]. The concentration of buildings is usually believed to cause an urban heat island (UHI) effect [10,11]. Buildings are obstacles that alter the interface of the wind ventilation and reduce the wind speed in the urban canopy layer. The building height, density and the shape of the street canyon determine the irregular influence of the ground surface, resulting in changes of solar radiation and turbulent diffusion, and urban ventilation capacity [12,13,14]. Buildings, streets, and other concrete constructions in urban areas absorb more solar radiation in diurnal periods and release heat slowly during nocturnal periods, resulting in UHI in building concentrated areas [15]. In turn, to capture this sort of local climatic variation, some comparable parameters are essential. The parameters, such as urban geometry, properties of surfaces, and vegetation, are very influential on the microclimate of these spaces. Remotely sensed land surface temperature (LST) has been widely used in the analysis of UHI at macro-scale [16,17]. Although the ground surface temperature is not equal to the urban air temperature, studies have proved that LST is closely related to near-surface temperature. LST can also be used to compare the relationship between UHI and the biophysical parameter [16,18,19]. A comparable topology has been designed known as the local climatic zone (LCZ) [20]. By analyzing the spatial characteristics such as surface structure (building/tree height and spacing), cover (pervious fraction), fabric (albedo, thermal admittance) and metabolism (anthropogenic heat flux), the urban spatial structure has obvious heterogeneity partitions and forms different local climate zones [21]. This research method of local climatic zone (LCZ) links urban heat state with the holistic urban morphology, and this sort of relationship is quite necessary for climatic adaptability of urban spatial planning [20].

At the site scale, many recent studies have analyzed the urban morphology that contributes to the development of UHI, and the microclimate of an urban street canyon has recently become an area of major concern. The term urban canyon (UC) replaced the primary structural unit in defining a common open space in an urban setting [22,23,24,25]. Canyon geometry can always be defined by several parameters, such as the basic value of the aspect ratio or height-to-width ratio (H/W), street orientation, the influence of Sky View Factor (SVF), and other descriptive indicators (building layout, and so on). Building orientation and street orientation can effectively improve the urban thermal environment [26,27,28]. Urban space height composition and agglomeration development mode have an important impact on residential daytime temperature [29,30]. The sky view factor (SVF) and building height have the greatest influence on the urban surface energy balance [11,31,32]. Different spatial patterns of the street boundary (building height, height to width ratio, cross-section composition) are closely related to the microclimate [10,33]. Canyon facades decrease monotonically as the canyon aspect ratio increases [34,35]. The optimal aspect ratio could assure an optimal microclimate, and the ideal aspect ratio for mid-latitude cities is 0.4–0.6 [36]. Applying an ideal H/W ratio for urban canyons from the averaged net radiative flux density would increase the wind speed by up to 35% and cool the air by up to 0.7 °C in Singapore using CFD (Computational Fluid Dynamics) [36]. Different architectural layout forms (terraced houses, enclosure layout, points group style) also have a heat island effect on the microclimate of residential areas [10,37]. Green patches, water bodies, and high albedo materials are very important for the functioning of summer temperature regulation [38,39,40,41,42,43].

To date, the study of the urban microclimate has largely shown a two-terminal distribution on the spatial scale, most of which are macro-scale or small-scale studies. On the macro-scale, this study primarily investigates the influence of urban underlying surface properties and morphological composition on urban local climate; on the micro-scale, the research objects are mostly focused on the simulation of the microclimatic effects of mutual group planning and layout and the study of wind environment characteristics in urban street canyons. On the medium spatial scale, the study of morphological indicators for identification in climatological research remains scarce, at least to an extent. A set of original morphological indicators of urban fabric into a porous medium with a rigid solid skeleton, including density, rugosity, porosity, sinuosity, occlusivity, compacity, contiguity, solar admittance, and mineralization, was previously proposed at the district or city scale [2]. Based on this indicators system, to study the relationship between urban morphology and air quality at the district level in Hong Kong, six variables (complete aspect ratio, occlusivity, roughness height, zero-plane displacement height, total building volume/number of buildings, and standard deviation of height) were proposed [44]. Based on an urban landscape model, a 2 km × 2 km area in Tokyo was used as a study site; the variables included surface area per projected area, volume per projected area, building to land ratio, mean height of buildings, surface area of buildings per unit volume of buildings and mean volume of buildings were used to feature morphological properties [45]. Most of the works applied the LCZ classification method for macro scale towns, but that method can also be applied for meso level towns with scale ranges from hundreds of meters to several kilometers [20,46]. Sky view factors (SVFs) and the sun-paths of urban canyons in an area of a medium-sized Brazilian city were studied by the development of maps in a GIS environment [47]. A range of multi-dimensional indicators were also proposed to reflect the relative influence of 2D and 3D indicators with land surface temperature [48].

The urban streetscape fabric morphology and the relationship to the microclimate environment formed under different historical backgrounds from the perspective of urban spatial planning are still not fully understood. The paper analyzes microclimate distribution under the influence of the street spatial morphology index, which belongs to four typical urban streetscape fabrics with four different historical backgrounds of urban succession development in Shanghai to provide optimization methods for climate-adaptive strategies of urban spatial development patterns and urban open space design on the medium scale.

## 2. Research Area and Methods

### 2.1. Study Area and Situation

In the historical period of the evolution of urban civilization in Shanghai, different historical backgrounds and development concepts have formed different settlement units and spatial zonings. The old Shanghai Town, which is considered the origin of Shanghai, was founded on a town built in the 17th century. This area played the role of city center in the evolution of urban functions and the variable development of spatial forms during different periods in Shanghai City. Following the initiation of Chinese reform, the Hongqiao Open Economic Zone was established, and the Gubei international community was constructed under the international impact of various cultures at the edge of the zone to meet the functional needs of living and international trade exchanges. The community zone provides another display of a “modern urbanization” area in Shanghai in the 1990s. In the 2000s, when many “city diseases” appeared in metropolitan cities because of pressure on the mono-centric city of the spatial structure, the suburban area of Shanghai was set up such that new residential towns could alleviate the congestion of the city center area. This area is well known as the “one city and nine towns” project of Shanghai. Anting New Town is the “modern” town representative of the “one city and nine towns” project, with features of a German style. In 2010, the four sub-center zones of the main city area in Shanghai were established, and the New Jiangwan Town came into being. The planning and design of New Jiangwan Town considered the natural skeleton of the watery regions blended with the international urban space, creating a new model town for local residents with a combination of local context and international standards. It is this overlapping of town partitions developed in different periods that created a cosmopolitan region of Shanghai with multiple cultures and urban vitality. In the process of the urbanization and industrialization of Shanghai, these town zones, which were formed at different times, evolved and have been continuously renewed.

**Figure 1:**
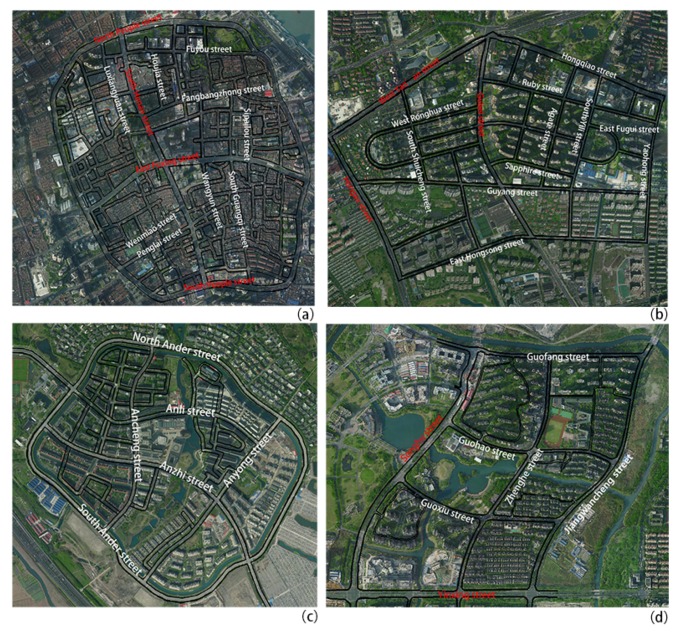
Satellite image and the streets fabric network of the four areas in 2017. (**a**) Old downtown zone, (**b**) Gubei international community, (**c**) Anting New Town zone (1.05 km^2^), (**d**) New Jiangwan Town zone (1.31 km^2^); Red fonts represent the urban main road; White fonts represent the branch road. Source: https://earth.google.com/web/.

Shanghai is located at the north latitude of 31°12′ and the east longitude of 121°30′ and is situated in the plain area of East China in the Yangtze River delta. Shanghai enjoys a subtropical monsoon climate, four distinct seasons, high temperature and abundant rainfall in summer and low temperature and less rainfall in winter. With global warming and climate change, extreme weather phenomena have been increasing for many years in Shanghai City, particularly extreme weather such as high temperature heat waves and heavy summertime rains. According to meteorological data of the Chinese Meteorological Administration Centre, extreme weather in Shanghai has appeared during the months of July and August, with the highest mean air temperature of the entire year being observed in early and mid-July. According to the statistical data of the Xujiahui general meteorological station, the frequency of the highest daily maximum air temperature (>35 °C) that occurred in 2017 peaked in July. In addition, there were 23 days with air temperatures above 35 °C. Based on the abovementioned large climate background, this study selected four representative urban fabrics in Shanghai as its research objects, including the Old downtown zone, the Gubei international community, Anting New Town zone, and New Jiangwan Town zone (Figure 1). The four zones appeared to have a different urban streetscape fabric from the rest of Shanghai because of their different historical backgrounds and planning concepts that created different spatial patterns. The date of this simulation was 23 July 2017.

The streetscape fabric of four urban spatial zones and their related indexes of spatial pattern in the regions are as follows (Figure 1 and Table 1):

(1) The old downtown zone is located in the central urban area of Shanghai with an area of 2.0 square kilometers, which has a unique spatial pattern of about 0.92 Floor Area Ratio (FAR) and high building density in the old housing blocks. The density of the street network is very high, the value reached 18.17 km/km^2^, and the streets are commonly narrow and curved. The average junction spacing is 98 m. There are 553 street segments and 184 neighborhoods [49]. Among these streets, the width of traffic corridors in the urban area can reach 40 m, the width of main streets is 7–8.5 m, and the width of each alley is 3–5 m. The buildings in this area typically have 2–4 floors with a Shanghai terrace housing style (a traditional Shanghai architectural style). The percentage of vegetation area is just 6.5%.
(2) The Gubei international community is located in Changning District, Shanghai, with an area of 1.34 square kilometers. This community’s block form is a typical enclosed courtyard style and has housing in rows in an east–west orientation, which constitutes two patterns of layout, namely, enclosure plan with peripheral open space and determinant plan. The buildings mainly comprise multi-story and high-rise buildings. The density of the street network is 8.69 km/km^2^, and has good traffic accessibility. The percentage of vegetation area is 31%, the distribution pattern of green space is of a scattered layout with large and small green patches [50].
(3) Anting New Town is located in Jiading District, west of Shanghai City. The new town covered an area of 5 square kilometers. For the scope of this study Anting New Town zone (the core area) covering 1.05 km^2^ in the west zone of Anting New Town is examined. The main neighborhood of the zone is surrounded by enclosure style, and the buildings have 4–5 stories each. The density of the street network is 7.80 km/km^2^, the pattern of street fabric is an irregular circle network layout. The width of the main street is from 28 meters to 40 meters, the inner street of the neighborhood is approximately 15 meters, and the street network and green corridors intersect in a network of open space [51,52], the percentage of vegetation area is 20%.
(4) New Jiangwan Town is located in Yangpu District, north of Shanghai City and covers an area of 9.45 square kilometers, for the scope of this study a Complete Lot of approximately 1.31 square kilometers was examined. Planning of the zone is primarily to create an ecological garden town [53]. The density of the street network is 6.42 km/km^2^, the pattern of street fabric is a declination grid pattern of a larger scale. The main neighborhood of the zone is mostly housing in rows in an east–west direction, and the buildings are composed of 5–6 multistorys and high-rise residential buildings with 11–17 stories each. The distribution pattern of green space is centralized large green patches combined with green networks, the percentage of vegetation area is 33%, the highest of the four study areas.

### 2.2. Research Methods

#### 2.2.1. Simulation Model and Data

**Figure 2:**
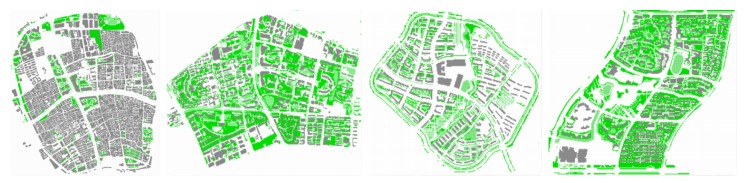
Modelling diagram of the four areas (From left to right: Old downtown zone, Gubei international community, Anting New Town zone, New Jiangwan Town zone).

ENVI-met is a three-dimensional non-hydrostatic microclimate model used to simulate the microclimate interaction of urban surface–vegetation–air under different meso-scale or micro-scale conditions [54]. The research scope of this paper is the urban microclimate at a medium spatial scale. The study area is in the range of 1 to 2 square kilometers. Version 4 of ENVI-met was adapted for this research to implement and input the simulation parameters in the setting process, i.e., the user-defined diurnal variation of the atmospheric conditions, finally enabling a realistic simulation [30]. The simulation date is 23 July 2017. The meteorological data came from the Weather Underground web site and the data of the Shanghai Hongqiao Airport weather station [55]. The initial input meteorological parameters are shown in Table 2, the air temperature value is the average values for the whole day of 23 July 2017. Because the coverage areas and characteristics of each zone are different, the spatial grid settings and parameter settings are also different. Detailed spatial parameter settings are shown in Table 3. The two-dimensional land use pattern of the simulated model of the four study area is shown in Figure 2.

#### 2.2.2. Street Morphological Indexes

On the urban meso-scale, the spatial morphological indexes were selected to describe the holistic microclimatic characteristics effects of the street system in the zone, such as the width, the orientation, the junction spacing, and the connectivity feature of the street network.

(1) The street width and orientation were used as common indexes to affect the microclimate factors. The influence of the different widths and orientations of streets on urban climate and environmental effects is directly related [56]. In Chinese traffic planning, street systems were divided into a main road system and a branch road system according to street grade. Normally, the widths of the main road were between 24 and 40 meters and the widths of the branch road were between 14 and 18 meters. The street orientation was divided into east–west and south–north.
(2) The junction spacing of each street was the average distance between the street junctions. The index of junction spacing could be used to reflect the density of the street network in a certain area [57], and also to present the spatial distribution of the number of junctions.
(3) Street connectivity was adapted to assess the degree of road network connectivity, which is calculated as the ratio of the actual number of links in the network to the maximum possible number of links in the network. The network connectivity of the street system is calculated as follows [58]:
  (1) $$\beta =\frac{2L}{V}$$
  where β is the connectivity coefficient, *L* is the number of links, and *V* is the number of nodes in the links network.

In terms of the influence of the effects of street site characteristics, the characteristic factors were analyzed from the three-dimensional spatial perspective, including Build-to-line ratio, Height-Width ratio (H/W), and Sky view factors (SVF).

The three-dimensional spatial indicators were expressed as follows:

(1) Aspect ratio (H/W): H/W reveals a negative linear correlation with the rate of decrease of air temperature in a given period [59].
(2) Build-to-line ratio (B): The build-to-line ratio is derived from the concept of the “street wall” in American urban planning [60]. It can be calculated as the ratio of the length of the frontage building near the street to the length of the red line of the street, and it is used to indicate the continuity of the building along the street. According to the statistical results of the index of the entire street network in Shanghai, there are five adapted classes in this study. T < 0.5 is the fifth-level control area; 0.5 < T < 0.6 is the fourth-level control area; 0.6 < T < 0.7 is the third-level control area; 0.7 < T < 0.8 is the second-level control area; T > 0.8 is a first-level control area.
(3) Sky view factor (SVF): SVF is a dimensionless parameter used to express the extent, for any point for a given location, to which a fraction of the overlying hemisphere is occupied by the open sky [59,60,61]. It is calculated by the ratio of the visible sky to the entire sky dome on the ground, which can characterize the ability of the ground in the study area to absorb solar radiation.

The width, Build-to-line ratio (B) and H/W values of the streets in the four study zones were obtained by measurement and calculation. The sky view factor (SVF) is calculated based on the simulation results of ENVI-MET software.

#### 2.2.3. Meteorological Variables and Thermal Comfort Index

To discuss the microclimatic condition of the street fabric in the four study areas, meteorological variables and the street thermal comfort index were used (namely, the air temperature (T), wind speed (WS), and Predicted Mean Vote (PMV)) to reflect the microclimatic characteristics of the street system and street ways. Predicted Mean Vote (PMV) is used for the thermal comfort index to express the difference in the heat stress between the human body and the environment. The data for each microclimate index in the region of each street were calculated by the mean values of the T, WS, and PMV values. Typically, the value of PMV is in the range of −4 to +4. When the value of PMV is in the range of −2 to +2, thermal comfort is better.

Three micro-climatic indicators—namely, air temperature difference (ΔT), wind speed difference (ΔWS), and PMV difference (ΔPMV)—were used in the correlation analysis of the micro-climatic effects of street three-dimensional spatial morphological indicators. These microclimate variable index factors were calculated by the typical street microclimate value minus the average value of the entire regional microclimate factor based on the simulation results of the four study zones. The difference algorithm was used to analyze the microclimatic differences of street space in different classification of the junction spacing and to analyze the influence of interface composition of each street morphology index on microclimate parameters on the street-site level.

The spatial structural and morphological characteristics of the entire streetscape fabric are formed by the changes in the interior nodes and the connectivity of the street system. When analyzing the corresponding relationship between the index of street connectivity and microclimate factors, the Coefficient of Variation (CV) data of temperature, wind speed and PMV in the internal street space were calculated, which could be used to analyze the dispersion degree of climate factors and thermal comfort data with different levels of street connectivity to reflect the fluctuation in the internal differences of these factors. Therefore, the CV values were used to present the fluctuation variation effect of microclimate indicators with the various values of street network connectivity at the meso-level. The formula for calculating Coefficient of Variation is as follows:

(2) $$\mathrm{CV}=\frac{SD}{MN}$$

where CV is the coefficient of variation; *SD* is the standard deviation; *MN* is the average of each index.

#### 2.2.4. Correlation Analysis between Street Morphological Index and Effects on Microclimate

The correlation relationship was analyzed according to the microclimate data corresponding to the different street morphology indexes of the four regions. The street system was divided into the main road and the branch road according to the different street widths in the four regions and combining the street orientations of east–west and north–south, the influence on microclimate factors of different scale levels were analyzed.

Hourly microclimate index classification data of the street width and different orientations were calculated to describe the relationship between holistic microclimatic characteristics effects and street system in the main period of the diurnal variation (from 6:00 to 15:00 in the simulated date) from the meso-level. The actual junction spacings were divided into different grade levels by calculating the junction spacing in the four study areas (Table 4). The various numerical values of hourly microclimate variation were analyzed by the grade designation, the air temperature difference (ΔT), and the wind speed difference (ΔWS) while PMV difference (ΔPMV) was used in the correlation analysis to directly reflect the microclimatic effects of the junction spacing. By calculating the connectivity of the actual street network system in four zones, the method is as in formula (1). The connectivity of the street network system in the old downtown zone is 4.7; that in the Gubei international community is 3.23; that in Anting New Town zone is 3.09; and that in New Jiangwan Town zone is 2.83. The CV values of the air temperature, wind speed and PMV values were calculated to analyze the microclimatic fluctuation of the street network system under different street connectivity data in the four zones; the method is as in formula (2).

### 2.3. Validation of ENVI-Met Software

**Figure 3:**
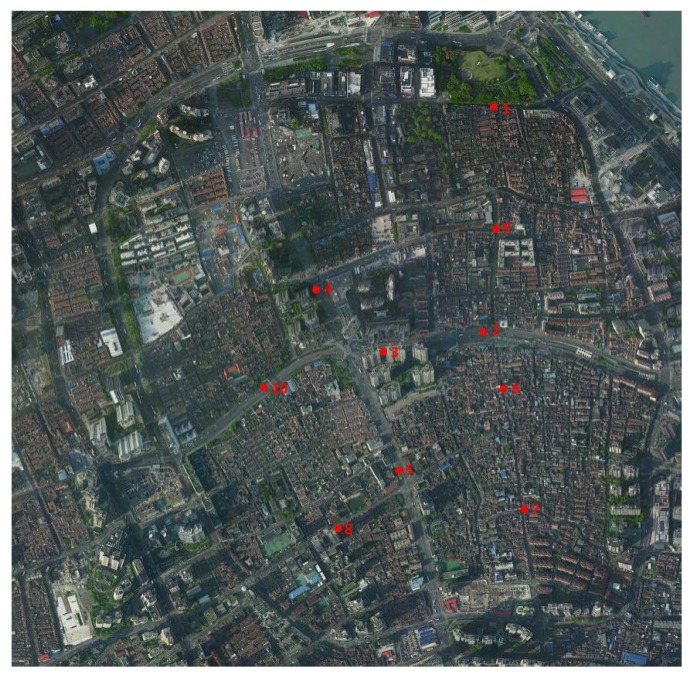
Monitoring points for field measurements in the old downtown zone values and simulated values.

The accuracy of the ENVI-met simulation is verified by comparing the results of simulated values with field measurements. The old downtown zone was selected as the site of measurement, and 10 monitoring points were arranged. Each monitoring point was measured separately five times, and we calculated the average value as the meteorological value of that point. For the monitoring points data (Figure 3), including different width levels of the street in different locations, hand-held meteorological instruments (kestrel 5000) were used to measure the meteorological situation at 1.5 m of the ground at 10:00 a.m. on 10 August 2018.

The verification method for the model used the Root Mean Square error (RMSE) method, which is an effective method to verify the deviation between measured values and simulation values. When the RMSE value is large, the error between the simulated and measured values is very large, and vice versa. This method is commonly used to verify the feasibility of climate simulation software. The formula for calculating the RMSE is as follows:

(3) $$\mathrm{RMSE}=\sqrt{\frac{\sum_{i=1}^{n}{\left(x_{i}^{\prime }-x_{i}\right)}^{2}}{n}}$$

where RMSE indicates the root mean square error value, $x_{i}^{\prime }$ indicates the observed value, $x_{i}$ indicates the simulated value, and *n* indicates the number of measured points.

**Figure 4:**
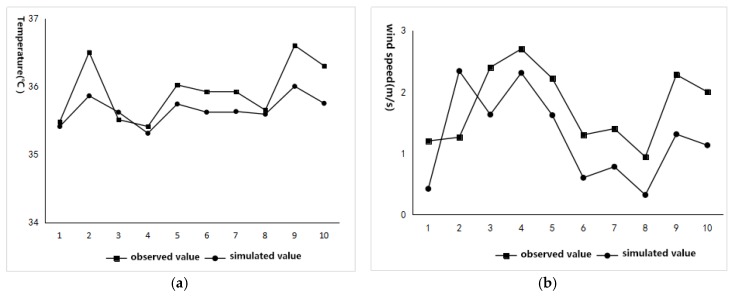
Comparison between measured values and simulated values for verifying the validity of Envi-met software. (**a**) Air temperature values; (**b**) wind speed values.

By comparing the measured and simulated values of 10 monitoring points (Figure 4), it is first seen that the variation trend between the measured and simulated temperatures and the wind speeds of the 10 monitoring points have a strong trend toward consistency. Second, by calculating the values of the Root Mean Square error (RMSE) between the measured and simulated points, the RMSE value of the air temperature is 0.7 °C, the Mean Absolute Percentage Error (MAPE) value of the air temperature is 8%; the RMSE value of the wind speed is 0.76, and the MAPE value of the wind speed is 8.9%. The RMSE model method shows that the error values between the measured values and the simulated values in the study are all small, and the prediction of microclimatic conditions can be considered to have a good precision [62,63].

## 3. Results and Discussion

### 3.1. Microclimate Factor Characters of Streetscapes Fabric Indicators in the Medium Spatial Zone

#### 3.1.1. Morphological Indexes of the Street Fabric and Their Air Temperature Distribution

(1) Street width and orientation

**Figure 5:**
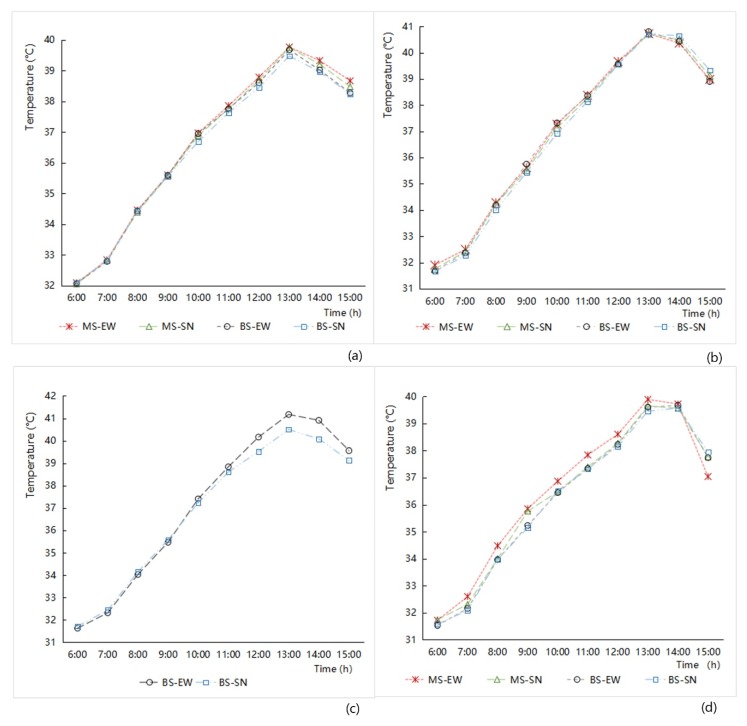
The hourly air temperature variation analysis related to different grade width and orientation of street space from 6:00 to 15:00 in the four study areas. (**a**) Old downtown; (**b**) Gubei international community; (**c**) Anting New Town; (**d**) New Jiangwan Town.

In terms of street width, the effect on air temperature showed that the average temperature of the main road network was higher than that of the branch road network. The analysis of the hourly temperature data variation showed that the air temperature in the street space increased hourly in the morning, reached a maximum at 13:00, and then decreased. The extent of the decline was affected by different environmental factors and showed variable changes (Figure 5). Under the same condition of street width, the air temperatures of the main road system in the old downtown zone were higher than those of the branch roads system at 6:00–15:00, and the differences steadily increased after 9:00 (Figure 5a). The air temperatures of the main road system were higher than those of the branch road system in the Gubei international community at most times, and the temperature differences decreased with the increase in solar altitude angles (Figure 5b). From 6:00 to 14:00 in New Jiangwan Town zone, the air temperature of the main road system was higher than that of the branch road system (Figure 5d).

With respect to the street orientation aspect, the air temperature of the street fabric along the south–north orientation was lower than that along the east–west orientation at most times. With the hourly variation of the solar altitude angles, the shadowing effect of buildings by the streets with a south–north orientation was produced. The roadway areas with an east–west orientation had relatively weak shadowing from solar radiation. Especially in the afternoon, the east–west streets are exposed to direct sunlight, which leads to relatively high street temperatures (Figure 5). The street orientation and air temperature characteristics of Anting New Town zone were consistent with the above mentioned law mainly after 9:00, but during the period from 6:00 to 9:00, the air temperature of the street space was slightly higher in the south–north street system than in the east–west street system (Figure 5c). This selected area has the characteristics of road inclination. In the morning, the solar altitude angles are small, producing different shadows from different angles, resulting in complex data variation in street temperatures with different orientations, and the regularity laws were not consistent. With the increase in the solar altitude angle, the influence of the solar radiation intensity increase promoted the former consistency law, again playing a leading role in air temperature distribution.

(2) Junction spacing

**Figure 6:**
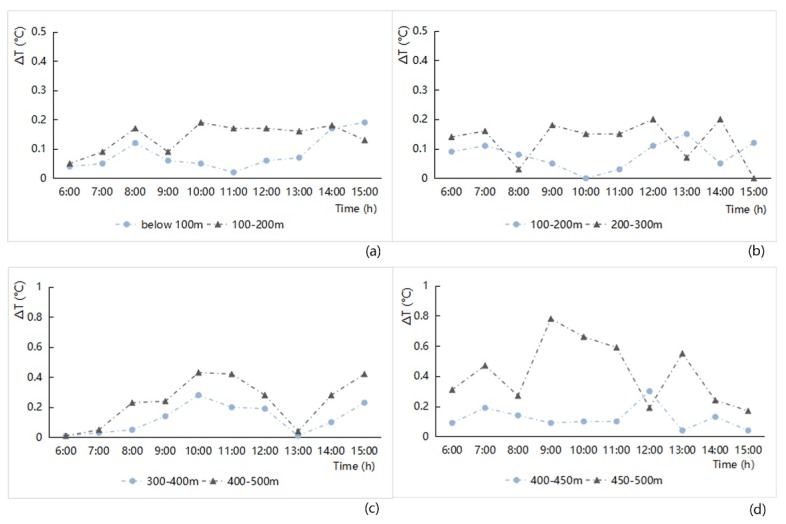
Hourly ΔT variation analysis related to different grade junction spacings of the street network from 6:00 to 15:00 in the four study areas ((**a**) Old downtown; (**b**) Gubei international community; (**c**) Anting New Town; (**d**) New Jiangwan Town).

The influence of the distance on the air temperature factor was analyzed by different grade levels of the junction spacing and correlating the designation levels with the hourly data variation in ΔT values in the streetway area. The result is shown in Figure 6. Overall, the larger the junction spacing between the streets, the greater the warming amplitude of the street network; the smaller the junction spacing between the streets, the denser the street network and the smaller the variation of the ΔT values. More specifically, when the streetscape network had open features, the air temperature changes in it were greater and the microclimate heating effect of the street network was prominent. When the streetscape network had dense features, the changes in its air temperature changes were smaller, and the influence of the surrounding features had a significant effect. Detail regularity could also be found as follows:

(a) The temperature difference (ΔT) of the street network in most areas followed the increase in the junction spacing.
(b) The ΔT values of the study zone increased relatively slowly before 9:00 then the ΔT values from 9:00 to 12:00, followed the increase in the junction spacing. When solar altitude angles were small, solar radiation was not very strong. With the hourly increase in solar radiation, the average temperature of the street space in the study zones increased rapidly. However, the increase data for the air temperature in the entire study zone was affected by the different building environments, and the average temperature of the study zone increased relatively slowly, which could explain why the ΔT values had the phenomena with the lowest point, at least to a certain extent;
(c) After 12:00, there was a low inflection point and a decreasing trend of ΔT values between the warming amplitude of the air temperature and the junction spacing of the street network. At noon, the solar altitude angles reached their maximum, and the street space and the entire study zone all had high temperatures. The ΔT values between them were very small. In particular, the shadow effect in the old urban area makes the ΔT values of the street network relatively stable because of the overly dense building coverage. The street network of Anting New Town zone has the characteristics of road inclination and bending curvature, and the change of ΔT values was relatively complex.

(3) Street connectivity

**Figure 7:**
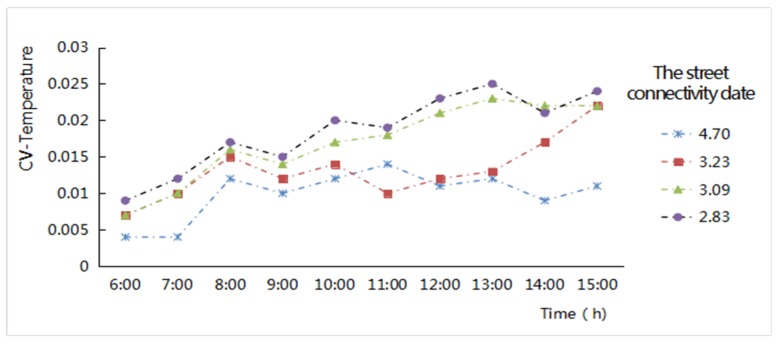
Hourly Coefficient of Variation (CV) values variation of the air temperatures related to the different levels of street connectivity of the street network from 6:00 to 15:00 in the four study areas.

The calculation of the CV values of the air temperature was adapted to present the discrete degree of the air temperature difference fluctuation in the roadway area. The indices values of street connectivity had clear differences among the four study zones. The minimum value is in New Jiangwan Town zone, at 2.83; the value continued to increase from Anting New Town zone to the Gubei international community, at 3.09 and 3.23 respectively; and the maximum street connectivity is in the old downtown zone, at 4.7. The relationship between different levels of street connectivity and the CV values of the air temperatures shows that (Figure 7), the larger the street network connectivity, the smaller the CV values of the air temperature in the streetway area. With increases in the solar altitude angle, the CV values in the street network hourly increased. Namely, the connectivity of the street network was very much denser, the air temperature distribution in the street network could be more uniform, and the fluctuation was very small. When the diurnal air temperature increased hourly, the temperature distribution inside the street network would be more uneven, and the fluctuation would be greater. Once it was affected by environmental factors, the relationship between street network connectivity and the discrete degree of the temperature factors would deviate from and disturb their regularity.

#### 3.1.2. Morphological Indexes of the Street Fabric and Their Wind Speed Distribution

(1) Street width and orientation

**Figure 8:**
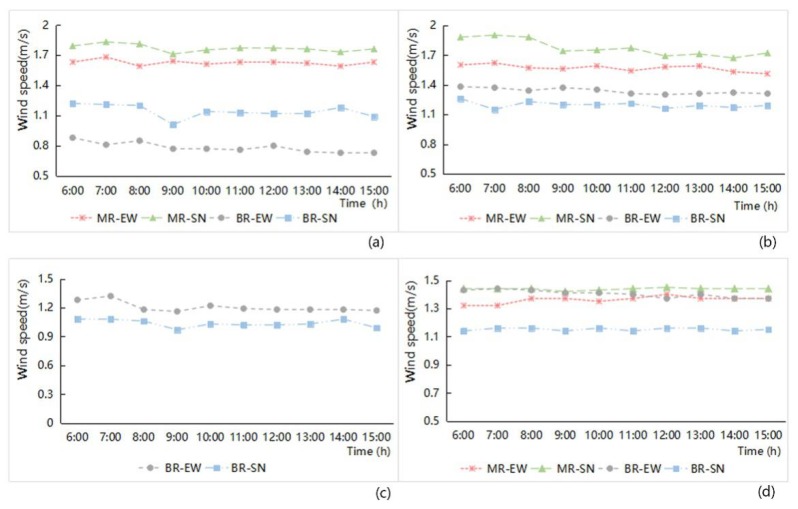
Hourly wind speed variation analysis related to the different grade width and orientation of street space from 6:00 to 15:00 in the four study areas ((**a**) Old downtown; (**b**) Gubei international community; (**c**) Anting New Town; (**d**) New Jiangwan Town).

In terms of street width, the effect of wind speed indicates that the average wind speed of the main road network was higher than that of the branch road network, as shown in Figure 8. The analysis of the hourly wind speed data variation showed that the wind speeds in the street space were basically stable and less affected by the variation of the solar altitude angle. The overall average wind speeds of the street network in Anting New Town zone and New Jiangwan Town zone were significantly lower than those of the old downtown zone and the Gubei international community, which reflects the effect of the street canyons created by streetscape elements. In a street space with strong enclosure substance, air fluidity was enhanced and wind speed increased as a result. Under the same condition of street width, the wind speeds of the main road system in the old downtown zone and the Gubei international community were all greater than those of the branch road, and there is a substantial difference in the wind speed of the main and the branch roads in the old downtown zone. In New Jiangwan Town zone, the wind speed of the main road with an east–west orientation was greater than that of a branch road with the same orientation. This special situation was also affected by the street canyon effect of different enclosed street spaces. At the same time, the angle inclination between the road direction and the wind direction in this zone conformed to the direction of the southeast monsoon, which was another important factor in creating the increased wind speed in the branch roadway areas.

From the street orientation aspect (Figure 8), under the same condition of the street width, we see that the average wind speed of the main road system with a south–north orientation was higher than that of the main road system with an east–west orientation; the average wind speed of the branch roads system with a north–south orientation was still higher than that of road with an east–west orientation in the old downtown zone. However, in the Gubei international community, Anting New Town zone, and New Jiangwan Town zone, the wind speeds of the branch roads with an east–west orientation were higher than the speeds of the branch road with a south–north orientation. This finding related to the angle inclination between the north–south streets and the direction of the southeast monsoon. The angle inclination of their north–south streets in the three study zones is partly or totally leeward, resulting in an overall wind speed of the north–south streets that was not very high; the angle inclination of the east–west streets in Anting New Town zone and New Jiangwan Town zone was downwind, and the wind effect of the street canyons in east–west streets was obvious by their enclosed buildings, which aggravated the wind speed.

(2) Junction spacing

**Figure 9:**
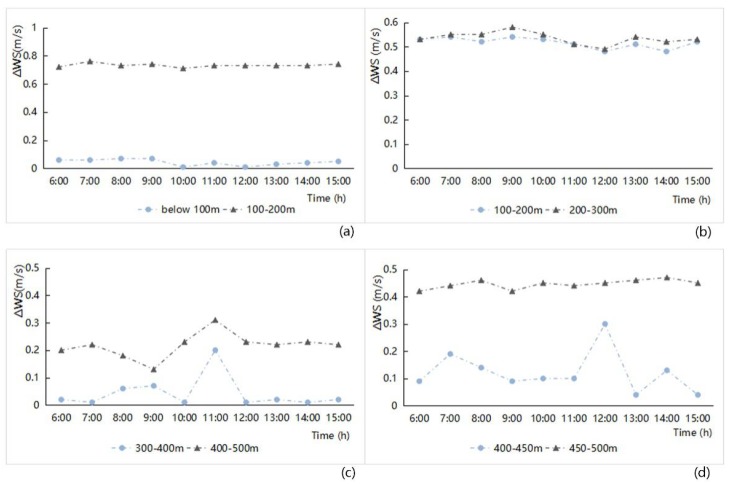
Hourly wind speed difference (ΔWS) variation analysis related to different grade junction spacings of the street network from 6:00 to 15:00 in the four study areas ((**a**) Old downtown; (**b**) Gubei international community; (**c**) Anting New Town; (**d**) New Jiangwan Town).

The influence of the junction spacing on the wind speed factor was analyzed using hourly variations in the ΔWS value of the street space to correlate the designation levels with the hourly data of values in the streetway area. The result is shown in Figure 9. The larger the junction spacing between streets, the larger the ΔWS value; the smaller the junction spacing, e.g., the streets in a spatial unit with high density, the smaller the variation in ΔWS value. More specifically, when the streetscape network had open features, the microclimate ventilation effect of the street network was prominent; When the streetscape network had dense features, the microclimate ventilation effect of the street network in it was bad, and the influence of the surrounding features was significant. Among the four study zones, in the old downtown zone, the junction spacing is especially small, while the junction spacing of several main roads is relatively large, resulting in the difference of ΔWS values between the two classes being particularly obvious. In the Gubei international community, the junction spacing was relatively small, the ventilation condition of the streets was inadequate, and the ΔWS value difference between different interval levels was the smallest (Figure 9). In view of the impact of junction spacing index on the wind speed, New Jiangwan Town zone had a large-scale street network layout, which embodied the best ventilation corridor effect.

(3) Street connectivity

**Figure 10:**
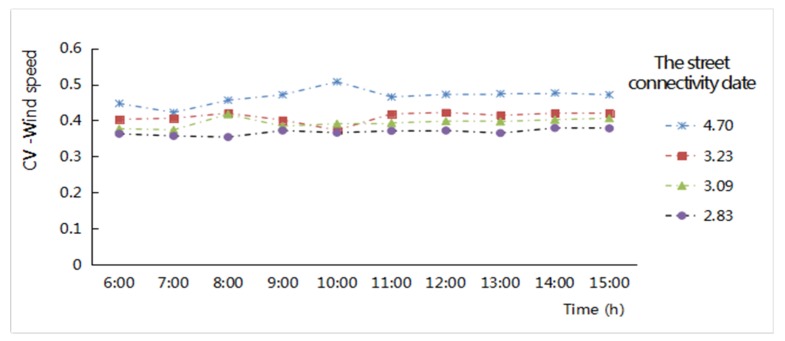
Hourly CV values variation of the wind speed related to different levels of street connectivity of the street network from 6:00 to 15:00 in the four study areas.

The CV values of the wind speed were used to reflect the discrete degree of spatial wind speed in each zone of the street network (Figure 10). There was a linear positive correlation between the CV values of the wind speeds and the street connectivity in the four study areas. The relationship between different levels of street connectivity and the CV values of the wind speed shows that with the increase in street connectivity, the CV difference in wind speed increased in the roadway area, which indicated that the street network had good connectivity and provided better traffic accessibility; the diversion of wind speed is more apparent in the intersection, which creates alteration and fluctuation of the wind speeds in different sections of the street network. With the increase of the solar altitude angle, the hourly variation of discrete degree CV values in the street network increases slightly, but the variation trend was very small. This feature showed that the ventilation conditions mostly affected the form and enclosure of the spatial composition of the streetscape fabric and were weakly affected by the alteration of solar radiation (Figure 10). According to the relationship between the street connectivity index and wind speed in the roadway area, the street network of New Jiangwan Town zone was relatively open, and its wind speed was stable. In the old downtown area, the street network had a large density and many nodes, the wind speeds in the roadway areas were uneven, and the air convection exchange was relatively frequent in the interior.

#### 3.1.3. Morphology Indexes of the Streetscape Fabric and the PMV Distribution

(1) Street width and orientation

**Figure 11:**
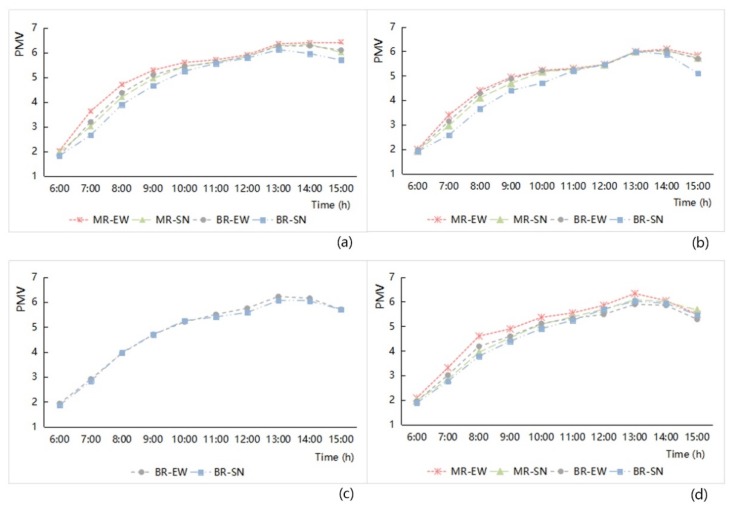
Hourly Predicted Mean Vote (PMV) variation analysis related to the different grade width and orientation of street space from 6:00 to 15:00 in the four study areas ((**a**) Old downtown; (**b**) Gubei international community; (**c**) Anting New Town; (**d**) New Jiangwan Town).

By analyzing the variation characteristics of hourly PMV values (Figure 11), we found that average PMV values of the different street classifications based on the width and orientation increased hourly in the morning, reached their maximum at 13:00–14:00, and then decreased. The extent of the decline was affected by different environmental factors and showed variable changes. The hourly change characteristics of PMV index were similar to those of air temperature.

In terms of the influence of street width, the average values of PMV of the main road network were generally higher than those of the branch road network. However, ventilation conditions are another factor affecting PMV. The ventilation ability of east–west streets was not as good as that of the north–south road; for that reason, the PMV value of the east–west road is higher than that of the south–north road. Together, the street width and orientation affect the distribution characteristics of PMV values. The specific impact analysis of the four research areas is as follows:

(a) The PMV values for the east–west streets in the old downtown zone and the Gubei international community were higher than those for the north–south streets. Under the same orientation, the PMV values on the main road were all higher than those of the branch road (Figure 11). This characteristic showed that the influence of street orientation on PMV factor was greater than the influence of street width.
(b) The study area of Anting New Town zone has only the branch road. Figure 11 shows that the PMV value of east–west streets was higher than that of north–south streets. The difference between them is not obvious; it is affected by the curvature of the street network and the inclination of street orientation.
(c) The PMV value of the main road with east–west orientation in New Jiangwan Town zone was higher than that of the south–north orientation. Between 6:00–12:00, the PMV value of the east–west orientation of the branch road was higher than that of the south–north orientation. From 12:00–15:00, the PMV value of the branch road with the south–north orientation was higher. The linear trend embodied the PMV index of the east–west streets was higher than that of the north–south road, which is largely in accordance with the general finding. The special distribution of the PMV values was affected by the morphological factor of the road inclination angle. At the same time, the shadows of the surrounding objects under the influence of the solar altitude angle were also an aspect of changing the streets’ thermal comfort.

(2) Junction spacing

**Figure 12:**
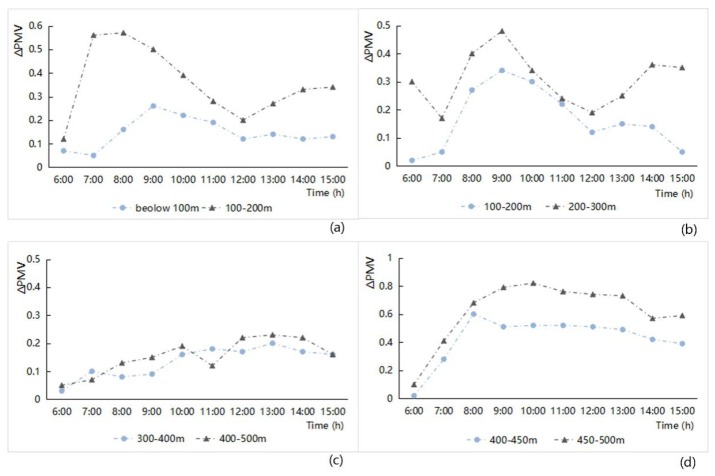
Hourly ΔPMV variation analysis related to different grade junction spacings of the street network from 6:00 to 15:00 in the four study areas ((**a**) Old downtown; (**b**) Gubei international community; (**c**) Anting New Town; (**d**) New Jiangwan Town).

The influence of the junction spacing index on the PMV factor was analyzed using the hourly variation of the ΔPMV value in the roadway areas to correlate with the effect of different levels of the junction spacing. The results are shown in Figure 12. Overall, the larger the junction spacing between streets, the greater the change in the PMV increment of the street network. More specifically, when the streetscape network had open features, the thermal comfort of the street network in it was poor; when the streetscape network had dense features, the thermal comfort perception of the street network in it was better, and the influence of the surrounding features was significant. The specific impact analysis of the four study zones was as follows (Figure 12):

(a) There are big differences in thermal comfort corresponding to the different grade of junction spacing in the old downtown zone. When the junction spacing was of higher-level, the ΔPMV value is bigger, and the thermal comfort perception of the street space was not good; The junction spacing was of low-level, the ΔPMV value is smaller, and the differences between the thermal comfort perception of the street space and the surrounding area was relatively small.
(b) The junction spacing in the Gubei international community and the old downtown zone all have smaller standard levels. The junction spacing distribution involved a relatively even distribution, and the influence of junction spacing on thermal comfort perception was also relatively small.
(c) The hourly changes in the ΔPMV value in Anting New Town zone were the smallest. Although the junction spacing was on a medium scale, it was also affected by the streets’ inclination, which conformed to the direction of the southeast monsoon, and the impact of street enclosure elements in the zones. The street network in the zone had the optimal thermal comfort perception.
(d) The junction spacing in New Jiangwan Town zone was relatively large, the ΔPMV value of the large-scale street network was at a maximum, and the thermal comfort perception of the street space in the zone was at its lowest.

(3) Street connectivity

**Figure 13:**
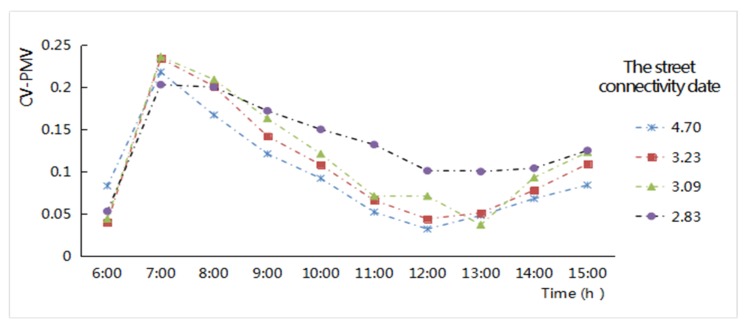
Hourly CV values variation of the PMV value related to different street connectivity of the street network from 6:00 to 15:00 in the four study areas.

The CV values of the PMV were used to reflect the discrete degree of PMV value in each zone of the street network. The hourly CV values of PMV for the main period from 6:00 to 15:00 were calculated as shown in Figure 13. According to the variation characteristics of the CV values of the PMV, the higher the street connectivity, the smaller the CV values of the PMV factors. This characteristic showed that the street network with good connectivity had a relatively even PMV distribution; the fluctuation in it was very small. With the increase in the solar altitude angle, in the period of strong solar radiation, the differences in the PMV discrete degree (CV value) in the street space decreases. More specifically, under nearly direct solar radiation or direct sunlight, the street space was less affected by shadows, the PMV values of all sections of the streets increased evenly, and the distribution of PMV values on the streets fluctuated slightly. In contrast, when the solar altitude angle is low, the PMV values of all sections of the streets were greatly affected by the characteristics of the surrounding substance, the distribution of PMV values of the street network was very uneven, and the CV values of PMV fluctuated considerably.

### 3.2. Morphology Index of the Streetscape Fabric and Microclimate Factor Distribution at the Street-Site Level

**Figure 14:**
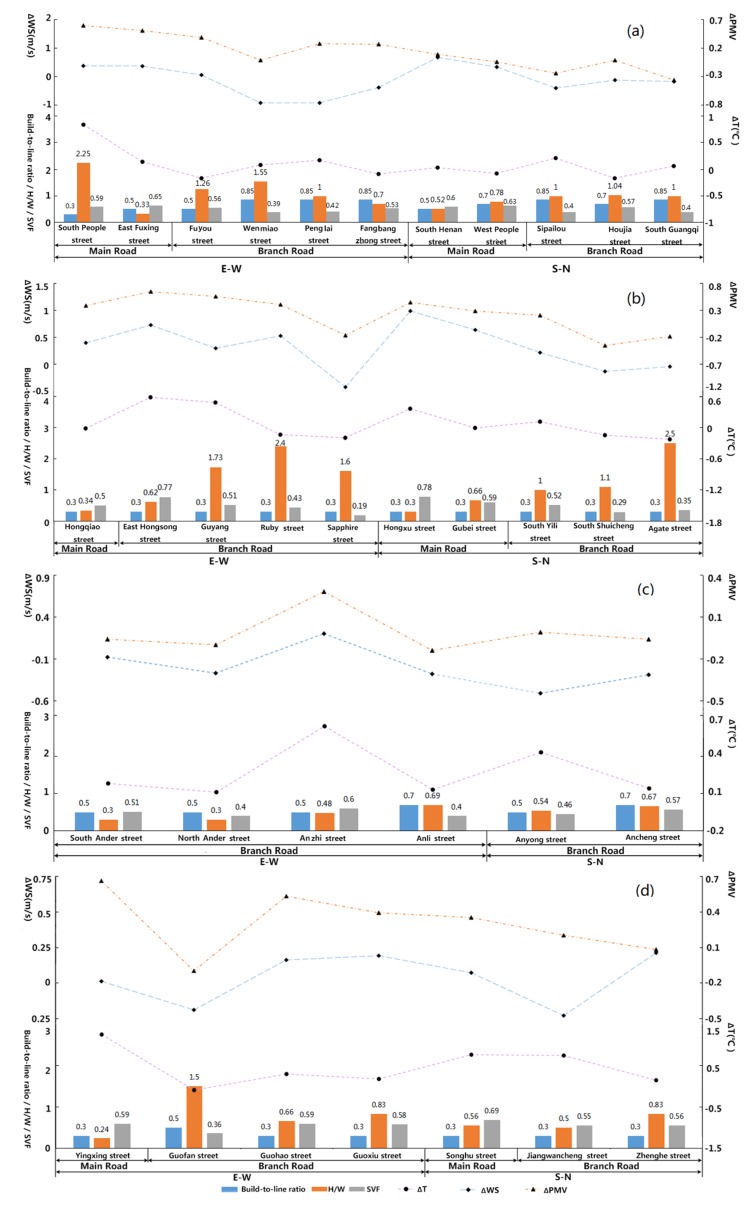
Spatial correlation comparison diagram of the four study zones between the morphological indexes and microclimate factors at street-site level for the four study zones ((**a**) Old downtown, (**b**) Gubei international community, (**c**) Anting New Town zone, and (**d**) New Jiangwan Town zone).

**Figure A1:**
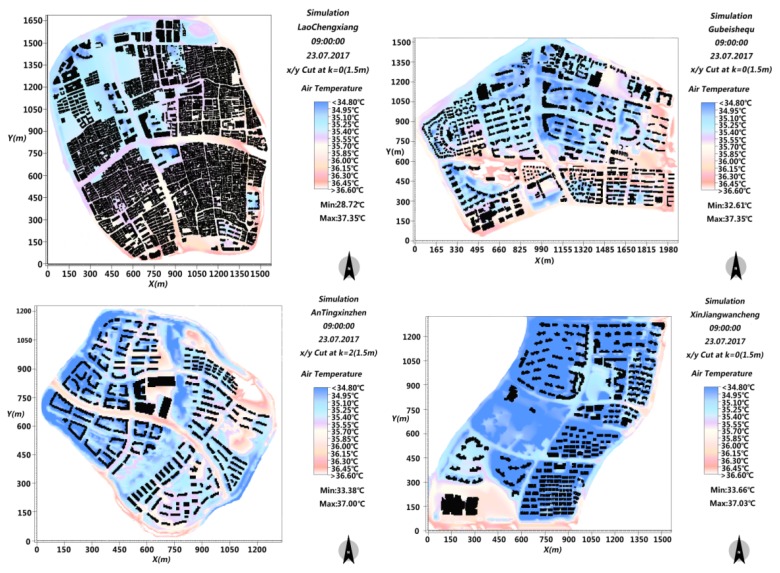
Spatial distribution of the air temperature in the four areas at 9:00 p.m., 1.5 m (**left upper**: Old downtown; **right upper**: Gubei international community; **left lower**: Anting New Town; **lower right**: New Jiangwan Town).

**Figure A2:**
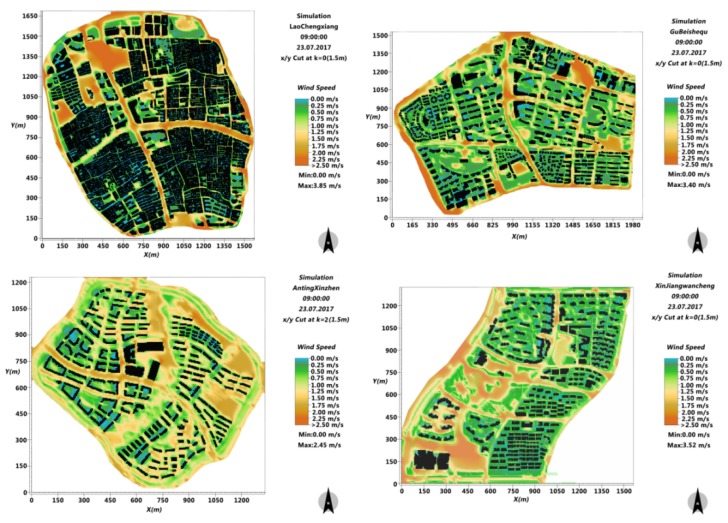
Spatial distribution of the wind speed in the four areas at 9:00 p.m., 1.5 m (**left upper**: Old downtown; **right upper**: Gubei international community; **left lower**: Anting New Town; **lower right**: New Jiangwan Town).

**Figure A3:**
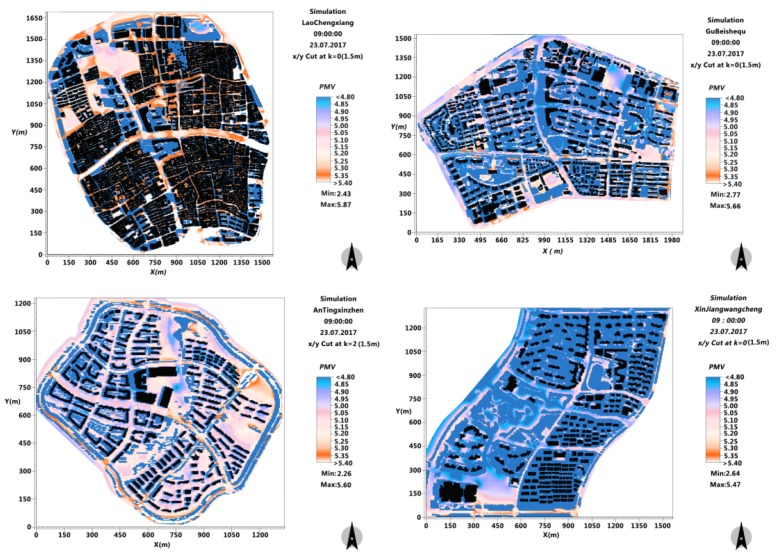
Spatial distribution of the PMV values in the four areas at 9:00 p.m., 1.5 m (**left upper**: Old downtown; **right upper**: Gubei international community; **left lower**: Anting New Town; **lower right**: New Jiangwan Town).

By choosing several typical streets through different classifications of street width and orientation in the four study zones, the linear correlation characteristics between their spatial morphological indexes (the Build-to-line ratio, Height-Width ratio, and Sky view factors) and the spatial distribution data of spatial microclimate factors in the specific segments were analyzed. The morphological indexes of spatial distribution on the street-site level were set using spatial dimensions, namely, the horizontal and vertical dimensions of the streetscape characteristics. The corresponding microclimatic effects of the various spatial form factors were compared and analyzed to discuss the detailed climatic adaptive methods. Considering the suitable time for residential outdoor activities, 9:00 a.m. was chosen for analyzing the microclimatic conditions of the streets’ fabric at a 1.5 m height off the ground. By comparing and analyzing the linear correlation results between the morphological indicators of different street classifications (road hierarchy and orientation) and air temperature in the four zones, the following spatial relationship characteristics of the morphological indexes’ microclimatic impact at streetscape space could be identified (Figure 14, Figure A1, Figure A2 and Figure A3).

#### 3.2.1. Correlation Characteristics between the Streetscape Morphology Index and Air Temperature

(1) Main roads with east–west orientation

These street segments of urban main roads had higher average air temperatures than the entire average temperature of the study zone. There is no main road in Anting New Town zone. The ΔT values in New Jiangwan Town zone (Figure 14d) and the old downtown zone (Figure 14a) were clearly higher than those of other streets in each of the same zones, indicating that the thermal environment of these zones would involve an urgent improvement area of good vegetation blocks or a centralized development area in a low-density pattern. South People Street in the old downtown zone (Figure 14a) had a very low Build-to-line ratio (below 0.5), its H/W value was relative higher in the zone, and the street had quite an open view; therefore, its SVF value was very high, and the ΔT value of this street segment was the largest. Yinhang Street in New Jiangwan Town zone (Figure 14d) had a low Build-to-line ratio, a low H/W value, a high SVF value, and its ΔT value was also very large. Comparing Yinhang Street, the H/W and SVF values were close, and East Fuxing Street (Figure 14a) in the old downtown zone had a relatively higher Build-to-line ratio (0.5–0.8), with the ΔT value being obviously lower. To some extent, these features reflected the index of the Build-to-line ratio affecting the air temperature change in the street space.

(2) Branch roads with east–west orientation

These street segments of the urban branches had higher average air temperatures than the entire average temperature of the study zone. The ranking of the average ΔT values is such that in Gubei Community it was the largest, followed by Anting New Town, and then that of in the New Jiangwan town, and the average ΔT value in the old downtown zone was the smallest. In the Anting New Town zone (Figure 14c), if the streets had similar values of SVF, the ΔT values of the street space were prominently influenced by the index of the H/W values. The higher the H/W value, the higher the ΔT value. If the streets had similar values of Build-to-line ratio (BL) and H/W ratio, this presented an increased ΔT value with higher SVF values. In the Gubei international community (Figure 14b), comparing the difference of air temperature between the Guyang street and Sapphire street, the two streets with similar BL values and H/W values, the higher the SVF value, the higher the ΔT value; in the old downtown zone (Figure 14a), the effect of the BL index on the ΔT value was obvious. The ΔT values of the streets with BL values above 0.8 were in entirety higher than that of the street with BL value of 0.5.

(3) Main roads with south–north orientation

These street segments of the urban main roads had higher average air temperatures than the overall average temperatures of the study zone. In general, the ΔT value of the north–south main roads was higher than that of the branchways in each of the same study areas. Because of the overly narrow branchway in the old downtown zone, there appeared some branches with a ΔT value that did not coincide with this feature. As mentioned above, the ΔT values of the north–south main roads were mostly lower than that of the east–west main roads. Gubei Street in the Gubei international community (Figure 14b) and Songhu Street in New Jiangwan Town zone (Figure 14d) had close lower values of the Build-to-line ratio and H/W; if the SVF value was higher, the ΔT value of the street space was correspondingly higher. Similar H/W and SVF values were found in South Henan Street in the old downtown (Figure 14a) zone and Songhu Street in New Jiangwan Town zone (Figure 14d); the Build-to-line ratio of South Henan Street was higher with a value of between 0.5 and 0.8, and the ΔT value appeared lower. Combined with the analysis of the characteristics of East Fuxing Street of the east–west urban main road in the old downtown zone, it can be seen that the Build-to-line ratio played a cooling role in a certain threshold range (the value of the study zones was identified between 0.5 and 0.8).

(4) Branch roads with south–north orientation

The average air temperature of the old downtown zone, Anting New Town zone and New Jiangwan Town zone is higher than the entire average temperature of the study zone. The ΔT values in the New Jiangwan Town zone were the largest, followed by Anting New Town zone, and in the old downtown zone it was the smallest. The ΔT values of this urban road hierarchy were negatively correlated with the H/W variables and positively correlated with the SVF variables (Figure 14). However, the SVF value of Houjia Street (Figure 14a) was higher than those of the other two branches in the old downtown zone, with the Build-to-line ratio calculated as between 0.5 and 0.8, and a low air temperature appeared on this street, which indicated that the effect of the Build-to-line ratio is more significant. The impact of the Build-to-line ratio was greater than that of the SVF index when the value was in the range of 0.5 to 0.8. Jiangwancheng Street and Zhenghe Street in New Jiangwan Town zone (Figure 14d) had the same Build-to-line ratio and SVF value, and the higher the H/W of the street, the lower the ΔT values. When the Build-to-line ratio and the H/W value were similar in the Gubei international community, the results showed that the higher the SVF value, the higher the ΔT value.

#### 3.2.2. Correlation Characteristics between Streetscape Morphology Index and Wind Speed

(1) Main roads with east–west orientation

These street segments of urban main roads had higher average wind speeds than the entire average temperature of the study zone. There is no main road in Anting New Town zone. The ΔWS value was relatively low and clearly lower than that of the south–north main roads. The ΔWS value of the east–west main roads in the old downtown zone is higher than that in the two other study areas, which demonstrated that the enclosure of the street building interface can promote the wind speed from the street canyon and alleviate the problem of the poor ventilation of the east–west streets. The Build-to-line ratio and the SVF value of South People Street in the old downtown zone (Figure 14a) were the same as those of Yinhang Street in New Jiangwan Town zone (Figure 14d). The H/W value of South People Street (Figure 14a) was higher, and the ΔWS value was correspondingly higher. The Build-to-line ratio and H/W value of Hongqiao Street in the Gubei international community (Figure 14b) and Yinhang Street in New Jiangwan Town zone (Figure 14d) were similar, and the SVF value of the Hongqiao Street was higher, with the ΔWS also being higher.

(2) Branch roads with east–west orientation

The wind speed of this type of urban branchway in the old downtown zone and Anting New Town zone was generally lower than the entire average wind speed of the study zone, and the ΔWS values were larger in the old downtown zone than in the Anting New Town zone. The wind speed of the streets in the Gubei international community and the New Jiangwan Town zone were higher than the entire average wind speed of the study zone, and the ΔWS values were larger in the Gubei international community than in the New Jiangwan Town zone.

The streets in New Jiangwan Town zone (Figure 14d) were relatively open and the wind speeds were relatively high, and the higher the H/W value, the smaller the ΔWS value. The lower the SVF value, the lower the wind speed. The characteristics are embodied in the case of the lower Build-to-line ratio and the higher H/W value of the Branch roads in the Gubei international community (Figure 14b). In Anting New Town zone (Figure 14c), when the Build-to-line ratio was the same, the wind speeds of this type of street were mainly affected by the H/W value and the SVF value of each streets, and the ΔWS value was greater when the H/W and SVF values of the streets were higher. The Build-to-line ratio was above 0.8, the ΔWS value was very low in the old downtown zone (Figure 14a), and the higher the H/W value of the street, the lower the ΔWS value. This outcome was mainly related to the narrower width of the street.

(3) Main roads with south–north orientation

The average wind speed of this type of urban main road was higher than the entire average wind speed of the study zone. The main influencing factors of the streets in the Gubei international community (Figure 14b) and New Jiangwan Town zone (Figure 14d) were the high SVF value of the street space caused by the high openness; the wind speed values of the main roads running from north–south were generally larger. The spatial distribution of wind speed in the old downtown zone (Figure 14a) essentially reflected that the impacts of the Build-to-line ratio and H/W index were very important. The higher the Build-to-line ratio and the H/W value of north–south main road was designed, the more the ΔWS values improved, and the street canyon effect was very significant in these cases. Comparing the simulation results of the three zones, it could also be concluded that with a more open street that embodied the higher value of the SVF, the effect of the SVF factor on ΔWS values was much greater than that of the Build-to-line ratio and H/W factors, and SVF had a greater influence on wind speed on the three-dimensional spatial scale vectors.

(4) Branch roads with south–north orientation

The wind speed values of this type of urban branchway were mostly lower than the entire average wind speed of the study zone. The ΔWS values of the north–south branches were mostly lower than those of the wider main roads, but higher than those of the east–west branchways of the same road hierarchy. The Build-to-line ratio and the SVF value of the branches in New Jiangwan Town zone (Figure 14d) were the same, and the higher the H/W value, the greater the ΔWS values. In the Gubei international community (Figure 14b), when the Build-to-line ratio and H/W value were similar, the higher the SVF values, the higher were the ΔWS values of the street. The same results were found in the old downtown zone; the Build-to-line ratio, the H/W value, and the SVF value in Anting New Town zone (Figure 14c) were all lower level, and the ΔWS values were also very low.

#### 3.2.3. Correlation Characteristics between Streetscape Morphology Index and PMV Value

(1) Main roads with east–west orientation

The ΔPMV values of this type of urban main road were higher than the entire average ΔPMV values of the study zone. The trend characteristics of the ΔPMV index were consistent with the regular pattern of the ΔT index, which was generally higher than that of other streets with other orientations. The PMV index was at its worst under the influence of the interface configuration in the street space of the old downtown zone, followed by New Jiangwan Town zone. This overall feature reflected that neither the renovation of the blocks interface in the old downtown zone nor the large-scale planning arrangement of the street space in New Jiangwan Town zone could form a superior thermal environment comfort for outdoor activities. When the Build-to-line ratio and the SVF value of Yinhang Street in New Jiangwan Town zone (Figure 14d) and South People Street in the old downtown zone (Figure 14a) were the same, the H/W value was higher, and the corresponding ΔPMV value was higher. The Build-to-line ratio of Hongqiao Street in the Gubei international community (Figure 14b) and Yinhang Street in the New Jiangwan Town zone (Figure 14d) were the same and very low, while the H/W value of Yinhang Street was lower than that of Hongqiao Street. However, the ΔPMV value of Yinhang Street is higher, which indicated the SVF value had a positive correlation with the ΔPMV factors in this type of street width.

(2) Branch roads with east–west orientation

In this type of urban branchway, the ΔPMV values of most streets in the old downtown zone, the Gubei international community, and New Jiangwan Town zone were higher than the entire average ΔPMV values of the study zone, while the ΔPMV values of streets in Anting New Town zone were generally lower than the average ΔPMV values of the study area. The ranking of the ΔPMV values was the old downtown zone > Gubei international community > Anting New Town zone > New Jiangwan Town zone. The branches in the old downtown zone (Figure 14a) had a higher Build-to-line ratio, and once the H/W value was higher, then the SVF value was lower, and the ΔPMV value was lower with good thermal perception for outdoor activities, which showed that the three-dimensional spatial vector has a positive impact on the thermal environment comfort in the case of a high Build-to-line ratio. Compared with other branches in the Gubei international community (Figure 14b), when the Build-to-line ratio and H/W were similar, the influence of SVF value appeared prominent. The lower the SVF value, the lower the ΔPMV value. The ΔPMV characteristics with different morphological indexes in New Jiangwan Town zone (Figure 14d) also reflected the fact that the H/W value of street space was negatively correlated with the three-dimensional space vector.

(3) Main roads with a south–north orientation

The ΔPMV values of this type of urban main road were higher than the entire average ΔPMV values of the study zone. Altogether, the ΔPMV values of the north–south main roads were higher than those of the branchways in the area. Because of the narrow width of Houjia Street in the old city (Figure 14a), it appeared that there was a high value distribution in the branchway, which did not conform to the former feature. Comparing the results of the four zones, it was found that the ΔPMV value was closely affected by the H/W and SVF indexes to some extent in this type of street, which was not obviously related to the Build-to-line ratio index. IF the H/W value of the street space was lower, its SVF value was higher, and the ΔPMV value of the street would be higher.

(4) Branch roads with south–north orientation

In this type of urban branchway, the ΔPMV values were lower than the overall average of the study zone in the old downtown zone, Anting New Town zone, and South Shuicheng Street and Agate Street in the Gubei international community, while the ΔPMV values in New Jiangwan Town zone and South Yili Street in the Gubei international community were higher than the overall average of the study zone. The average ΔPMV values of this type of urban branchway in the old downtown zone were the worst. When the Build-to-line ratio and H/W value of the branches in the old downtown zone (Figure 14a) were similar, once the SVF value was higher, the ΔPMV value was correspondingly higher. The Build-to-line ratios of South Yili Street and Agate Street in the Gubei international community (Figure 14b) were similar, and the street with a high H/W value had a lower ΔPMV value. The Build-to-line ratios of Anyong Street in Anting New Town zone (Figure 14c) and South Shuicheng Street in the Gubei international community (Figure 14b) were similar, Anyong Street having a higher H/W value, a lower SVF value, and a lower ΔPMV value. The Build-to-line ratio and the SVF value of Jiangwancheng Street and Zhanghe Street in New Jiangwan Town zone (Figure 14d) were the same, i.e., the street with the higher H/W value had the lower ΔPMV value.

## 4. Conclusions

By using a three-dimensional numerical simulation method, the microclimatic effects of the streetscape fabric were compared and analyzed with four Medium Spatial Zones formed in different historical contexts in Shanghai City, East China. The street network of the old downtown zone had the worst fabric microclimate environment, the average air temperature of the streets was the highest, the average wind speed of the streets was the worst, and the average PMV was the worst. Following was the Anting New Town zone, where the average air temperature of the streets was similar to that of the Gubei international community, but the average wind speed of the street network space was significantly lower than that of the Gubei international community, while the average PMV value of the thermal comfort index was also higher than that of the Gubei international community; therefore, the overall microclimatic conditions were worse than in the Gubei international community. The microclimatic condition of New Jiangwan City was optimal; the average air temperature of the streets was the lowest, the average wind speed was the largest, and the average PMV value was the smallest.

The morphological indexes of the urban street network, street width, street orientation, the junction spacing, and the connectivity feature of the street network all affect the microclimate factors of the streetscape fabric. The main roads always exhibited good ventilation, but the air temperature and PMV value were higher than those of the branch roads, and the overall microclimatic effects were not good; the south–north orientation always exhibited better microclimatic effects than those along the east–west orientation. The larger the junction spacing, the worse the air temperature and PMV values, but the better the ventilation effect of the street network. The street network with better connectivity should exhibit relatively better microclimatic effects, the fluctuation of wind speed was relatively larger, which was conducive to internal air circulation and strengthened the ventilation effect by forming urban street canyons.

At the street-site level, the spatial morphological indexes (the Build-to-line ratio, Height-Width ratio, and Sky view factors) of specific street spaces and the corresponding spatial distribution data of spatial microclimate factors were analyzed. The Build-to-line ratio of streets was closely related to the air temperature. When the value of the Build-to-line ratio was below 0.5 or above 0.8, the air temperature condition of the street space was not better, and once the value of the Build-to-line ratio was between 0.5 and 0.8, the street space generally had a positive relationship with the air temperature. The impact of the Build-to-line ratio was greater than that of the SVF index when the value was in the range of 0.5 to 0.8. The index of H/W and SVF had obviously different effects on the microclimate distribution of the street space. Street spaces of a given width—high value area of H/W and low value area of SVF—were basically the worst areas of microclimate factors. When streets were wider, such as the main roads, the influence of street width on microclimatic effects was greater than that of the impact from the H/W index. The SVF value of the streets was positively correlated with the wind speed, air temperature, and PMV value, while the H/W values were negatively correlated with wind speed, air temperature, and PMV value. Medium scale morphological indicators describing the street network also had a microclimatic impact on each street, which was embodied in a synergistic effect of the morphological indicators.

Applying the results of microclimate efficiency with different streetscape fabric and morphological structures in this study, the following optimization strategies of reducing the urban heat island intensity of the urban street network should be taken into account. (1) In terms of the overall pattern of the street network, a small junction spacing was conducive to mitigation of the air temperature heating intensity in the street space and improving the ventilation effect, reflecting that a small-scale neighborhood pattern can improve the microclimate of the entire regional street network better than large-scale neighborhood planning. (2) Within the network, street orientation has a significant impact on the distribution characteristics of air temperature, wind speed, and PMV. The north–south streets conforming to the direction of the Southeast monsoon in the study area were better than the east–west streets. Therefore, the street layout should be effective in directing the summer monsoon. The east–west streets have negative microclimatic effects, which can properly consider the streets’ inclination at a certain angle or in combination with a given curvature, reasonably guiding the summer monsoon entering the street space and improving the thermal and ventilation environment. (3) More street connectivity could create substantial fluctuations in the microclimate factors, which is conducive to internal air circulation and can benefit the ventilation effect by using the street canyons.

A comparative analysis of the microclimatic characteristics of the four medium scale zones provides obvious suggestions for urban design. Shanghai is in the stage of mature urbanization; this kind of city has multiple spatial fabrics. The street network of the old downtown zone represents the traditional life-style street space model, with very small junction spacings and an obvious street canyon effect. However, in the process of inheritance succession and protection development, the overall street network fabric faces regeneration needs, and the environmental comfort of its open space is in urgent need of improvement. According to the combination effects of spatial morphological indexes, these results showed that the rational layout of open space and strengthening the connectivity of the inner space in the urban old downtown zone play an important role in improving microclimatic conditions. Compared with the spatial form of Anting New Town zone and the Gubei international community, the street network layout of the Gubei community is more conducive to improving the microclimate of street space, with relatively small junction spacings between the streets and the interface of a street canyon. As a typical low-density residential area of Anting New Town zone with German features, the microclimatic conditions of the street fabric were not better. The differences in native natural and climatic conditions caused the alien town pattern style not to offer development adaptable to climate change in Shanghai, which has high-temperature weather in summer. The study zone of New Jiangwan Town zone is representative of an excellent garden eco-town in the mature stage of urbanization construction in China. In terms of mitigating the intensity of the heat island, the integrated development of the street fabric and internal open space in this area fully created a cooling effect throughout the region. However, the large-scale streetscape fabric is not the ideal pattern in terms of wind speed and thermal comfort.

The weakness of this research lies in the following. First, a detailed study on the street network indexes of different characteristic zones and deeper correlation characteristics between the streetscape morphology indexes should be performed. Second, the green space, squares, and other buildings enclosing open space in the study area should be further combined in a holistic open space system. This study has several limitations. First, the study only highlights a comparative analysis relying on the linear correlations of the street fabric to their thermal and ventilation indexes, which show a significant diurnal variation in summer. It would be very significant to mitigate urban heat island in cities that have high summer temperatures. Further studies are needed to account for observational errors (such as seasonal differences or thermal anisotropy) in the urban fabric. In general, this research method is worth introducing and applying to more medium-size quantitative researches. This possibility suggests that multispectral and multiresolution remote sensing images combined with GIS analysis can be combined with the study process of the in-depth comparative analysis.

## Figures and Tables

**Table 1:** The status of the building environment and streetscape fabric in the four zones.

	Building Time	Area (km^2^)	Road Density (km/km^2^)	Average Junction Spacing (m)	Percentage of Vegetation Area (%)
Old downtown zone	17th century	2.0	18.17	98	6.5
Gubei international community	1990s	1.34	8.69	240	31
Anting New Town zone	2000s	1.05	7.80	377	20
New Jiangwan Town zone	2010s	1.31	6.42	460	33

**Table 2:** Initial input values of weather parameters for the simulation model.

Input Parameters	Temperature (°C)	Wind Orientation	Wind Speed (m/s)	Humidity (%)	Roughness
Value	34.79	135°	3	75	0.01

**Table 3:** Parameters settings of the spatial models in the four study zones.

Simulation Parameters Setting	Area			
	Old Downtown Zone	Gubei International Community	Anting New Town Zone	New Jiangwan Town Zone
Number of grids (x,y,z)	525,563,16	375,306,15	267,246,35	313,265,13
Size of grid cell (m) (dx,dy,dz)	dx = 3,dy = 3,dz = 3 (base height)	dx = 5.5,dy = 5,dz = 3 (base height)	dx = 5,dy = 5,dz = 3 (base height)	dx = 5,dy = 5,dz = 3 (base height)
Number of nesting grids	3	3	3	3
The material for nesting grids	Soil A: Asphalt Soil B: Concrete	Soil A: Asphalt Soil B: Concrete	Soil A: Asphalt Soil B: Concrete	Soil A: Asphalt Soil B: Concrete
Default wall material	Concrete	Concrete	Concrete	Concrete
Default roof material	Tile	Concrete	Concrete	Concrete

**Table 4:** Grade level of junction spacings in the four zones.

Study Zones	Grade Level of the Junction Spacings
Old downtown	below 100
	100–200
Gubei international community	100–200
	200–300
Anting New Town	300–400
	400–500
New Jiangwan Town zone	400–450
	450–500
